# The Tissue Distribution of SARS-CoV-2 in Transgenic Mice With Inducible Ubiquitous Expression of hACE2

**DOI:** 10.3389/fmolb.2021.821506

**Published:** 2022-01-18

**Authors:** Alexander A. Dolskiy, Andrey S. Gudymo, Oleg S. Taranov, Irina V. Grishchenko, Ekaterina M. Shitik, Dmitry Yu Prokopov, Vladislav O. Soldatov, Elvira V. Sobolevskaya, Sergey A. Bodnev, Natalia V. Danilchenko, Anastasia A. Moiseeva, Polina Y. Torzhkova, Yulia A. Bulanovich, Galina S. Onhonova, Elena K. Ivleva, Marina V. Kubekina, Andrey E. Belykh, Tatiana V. Tregubchak, Alexander B. Ryzhikov, Elena V. Gavrilova, Rinat A. Maksyutov, Alexey V. Deykin, Dmitry V. Yudkin

**Affiliations:** ^1^ State Research Center of Virology and Biotechnology “Vector”, Rospotrebnadzor, World-Class Genomic Research Center for Biological Safety and Technological Independence, Federal Scientific and Technical Program on the Development of Genetic Technologies, Koltsovo, Russia; ^2^ Center for Precision Genome Editing and Genetic Technologies for Biomedicine, Institute of Gene Biology, Russian Academy of Sciences, Moscow, Russia; ^3^ Laboratory of Genome Editing for Veterinary and Biomedicine, Belgorod State National Research University, Belgorod, Russia; ^4^ Research Institute of General Pathology, Kursk State Medical University, Kursk, Russia

**Keywords:** SARS-CoV-2, intranasal infection, transgenic mice, hACE2 switch-on mice, ACE2, Cre recombinase

## Abstract

The novel coronavirus disease COVID-19 has become one of the most socially significant infections. One of the main models for COVID-19 pathogenesis study and anti-COVID-19 drug development is laboratory animals sensitive to the virus. Herein, we report SARS-CoV-2 infection in novel transgenic mice conditionally expressing human ACE2 (hACE2), with a focus on viral distribution after intranasal inoculation. Transgenic mice carrying *hACE2* under the floxed STOP cassette [(hACE2-LoxP(STOP)] were mated with two types of Cre-ERT2 strains (UBC-Cre and Rosa-Cre). The resulting offspring with temporal control of transgene expression were treated with tamoxifen to induce the removal of the floxed STOP cassette, which prevented hACE2 expression. Before and after intranasal inoculation, the mice were weighed and clinically examined. On Days 5 and 10, the mice were sacrificed for isolation of internal organs and the further assessment of SARS-CoV-2 distribution. Intranasal SARS-CoV-2 inoculation in hACE2-LoxP(STOP)×UBC-Cre offspring resulted in weight loss and death in 6 out of 8 mice. Immunostaining and focus formation assays revealed the most significant viral load in the lung, brain, heart and intestine samples. In contrast, hACE2-LoxP(STOP) × Rosa-Cre offspring easily tolerated the infection, and SARS-CoV-2 was detected only in the brain and lungs, whereas other studied tissues had null or negligible levels of the virus. Histological examination revealed severe alterations in the lungs, and mild changes were observed in the brain tissues. Notably, no changes were observed in mice without tamoxifen treatment. Thus, this novel murine model with the Cre-dependent activation of hACE2 provides a useful and safe tool for COVID-19 studies.

## Introduction

A recent outbreak of the novel coronavirus disease (COVID-19), which spread rapidly worldwide in just a few months, has become one of the most socially significant infections, along with influenza and smallpox (McEntire et al., 2021). The causative agent of COVID-19 is a positive single-stranded RNA virus of Coronaviridae found in the genus Betacoronavirus, which also includes severe acute respiratory syndrome-related coronavirus (SARS-CoV) and Middle East respiratory syndrome coronavirus (MERS-CoV) (with 80 and 50% nucleotide homology, respectively) (Kim et al., 2020). The diameter of viral particles corresponds to a wide range of particle sizes described in the literature, 60–140 nm, and the range of values can be explained by differences in the measurement methods used (Laue et al., 2021). Mature virions include several types of protein molecules divided into structural proteins [specifically, nucleoprotein (N), envelope protein (E), transmembrane glycoprotein (M), and glycoprotein (S)] and nonstructural proteins (including RNA-dependent RNA polymerase) (Kim et al., 2020). The interaction of the SARS-CoV-2 virus with the ACE2 cell surface receptor is mediated through the S glycoprotein embedded in the envelope. In most cases, this S-protein is cleaved by host proteases into S1 and S2 subunits, which are responsible for receptor recognition and membrane fusion, respectively (Lu et al., 2015; Zhang et al., 2020). Human tissue studies have shown an abundant presence of ACE2 receptors in the epithelia of the lung, small intestine, arterial and venous endothelial cells and arterial smooth muscle cells in all organs studied, including the brain (Hamming et al., 2004).

COVID-19 is characterized by high contagiousness and the development of complications that pose a threat to life. During the first days of infection, SARS-CoV-2 in the upper respiratory tract replicates without severe clinical symptoms. A few days after infection, metabolic changes occur in the cells, leading to the apoptosis of alveocytes and the possible occurrence of acute respiratory distress syndrome (ARDS). Infiltration of the lung tissue by rod-nuclear leukocytes leads to an increase in the secretion of proteolytic enzymes, which, together with the kallikrein-kinin system, the hemocoagulation system and the activation of components of the complement system, lead to secondary alteration of the lung tissue and expand the area of damage (Fan et al., 2020; Hanff et al., 2020; Lipcsey et al., 2021). Neurological symptoms of SARS-CoV-2 infection might be associated with systemic inflammatory response syndrome, sepsis, multiorgan failure or postinfectious, immune-mediated complications when the organism responds to the infection. It is believed that this virus can also affect the central nervous system directly or indirectly. Some neurological symptoms, such as encephalitis and anosmia–hyposmia, might be caused by the direct invasion of the central nervous system by the virus (Natoli et al., 2020; Pezzini and Padovani, 2020; Liu et al., 2021).

The pathophysiological patterns of the disease can only be studied by replicating tissue-specific and systemic virus-host interactions. Among the main models for studying COVID-19 pathogenesis and anti-COVID-19 drug development are sensitive laboratory animals. Animal models susceptible to infection with the SARS-CoV-2 virus include lower primates, hamsters, ferrets and mice (Takayama, 2020). The main criterion for the selection of model animals is sensitivity to the virus, which is directly proportional to the degree of conformity of the amino acid sequence of ACE2 in humans and animals. Thus, different homologs of the ACE2 receptor can also lead to fixation of mutations in the virus genome (Damas et al., 2020). The limitations of the use of animal models for certain studies of the virus may be due to an important feature. RNA viruses such as SARS-CoV-2 tend to evolve rapidly, which is associated with a high mutation rate. This genetic variability is modulated by both internal processes and external conditions. Among the internal factors, one can single out the lack of a corrective function of viral RNA-dependent RNA-polymerase in the process of replication, together with spontaneous mutations due to physical and chemical mutagens, which can also be harbored in the genome (Benedetti et al., 2020). External conditions that lead to a change in the virus include variants of receptors with which it interacts in the host organism at the cellular level. However, upon exposure to nonhuman variants of *ACE2*, the receptor that allows SARS-CoV-2 to enter cells, SARS-CoV-2 may change its properties due to an adaptation process that can lead to virus mutation (Welkers et al., 2021). To reduce SARS-CoV-2 variability and gain the ability to learn details of human SARS-CoV-2 infection, a few transgenic humanized mouse strains with hACE2 (human *ACE2*) expression have been produced.

This model constitutively expresses the hACE2 protein during embryogenesis and ontogenesis (Jiang et al., 2020; Sun et al., 2020). However, the constitutive expression of hACE2 sensitizes the model to SARS-CoV-2, while the mice are maintained in an animal facility or during transportation: these animals are accessible to technicians who may be infected with COVID-19 during the pandemic. This results in a risk of model animals being infected and producing antibodies, which may lead to incorrect conclusions during experiments involving SARS-CoV-2.

To avoid such difficulties during the research process, a conditional hACE2 switch-on mouse model was created and characterized by conditional expression of hACE2 (Bruter et al., 2021). The basic hallmark of this strain is the ability to spatially and temporally control hACE2 expression provided by the tamoxifen-induced Cre-recombinase-LoxP (STOP) machinery (Cre-ERT2). In brief, Cre recombinase is an enzyme that catalyzes site-specific recombination between two LoxP sites, resulting in the deletion of the LoxP-limited sequence (Kim et al., 2018). Thus, if the strain is mated with Cre mice, Cre recombinase is expressed. After tamoxifen administration, Cre recombinase gains access to the nuclear compartment and removes the STOP cassette, which prevents the expression of STOP-limited hACE2. Another hallmark of this model is that hACE2 is coexpressed with green fluorescent protein (GFP), making it possible to detect the levels of transgene expression without immunostaining or other complex protocols.

In the present work, we aimed to examine the SARS-CoV-2 sensitivity of the F1 generation obtained after crossing hACE2 (LoxP-Stop) with mice expressing tamoxifen-dependent Cre recombinase (Cre-ERT2). Herein, we report severe and mild SARS-CoV-2 infection in our model depending on which *Cre-ERT2* strain was utilized for transgene activation.

## Materials and Methods

### Animals

All animal procedures were carried out on 34 CBAxC57Bl6J mice of both sexes in accordance with the European Convention for the Protection of Vertebrate Animals Used for Experimental and Other Scientific Purposes (CETS No. 123). The experimental design was approved by the Ethical Committee of SRC VB “Vector” Rospotrebnadzor (protocol # 2 GNC VB VECTOR/02-04.2021, May 28, 2021). Groups of hACE2-harboring transgenic mice were obtained by breeding previously described hACE2(LoxP-Stop) mice (Bruter et al., 2021) and two strains of Cre-ERT2-mice: Ndor1^Tg(UBC−cre/ERT2)1Ejb^ and Gt(ROSA)26Sor^tm1(cre/ERT2)Tyj^ (Jackson Laboratory, United States). The Ndor1^Tg(UBC−cre/ERT2)1Ejb^ strain is characterized by the expression of the Cre/ERT2 gene under the control of the UBC promoter. This transgene is integrated into chromosome 2 at the Ndor1 locus. The Gt(ROSA)26Sor^tm1(cre/ERT2)Tyj^ strain is characterized by the expression of the Cre/ERT2 gene under the control of the CAG promoter. This transgene is integrated into chromosome 6 at the Rosa26 locus. Both strains are characterized by the nontissue-specific ubiquitous expression of Cre-ERT2 (Ruzankina et al., 2007; Ventura et al., 2007). Tamoxifen administration was carried out according to a previously published article (Bruter et al., 2021).

Hereinafter, hACE2(LoxP-Stop)✕Ndor1^Tg(UBC−cre/ERT2)1Ejb^ offspring are referred to as UBC-*ACE2,* and hACE2(LoxP-Stop)✕Ndor1^Tg(UBC−cre/ERT2)1Ejb^ offspring are referred to as Rosa-*ACE2*. Cre mice were genotyped according to protocols #27737 and #31150 provided by Jackson Laboratory with the forward 5′-AAA​CTG​TTC​AAT​ATG​CTG​AGG​CT-3′ and reverse 5′-AGT​AGT​TGA​GCA​GTG​GCC​TTA​C-3′ primers for hACE2 carried on the DNA cassette.

According to the experimental design, animals were divided into 7 groups: 1) infected UBC-*ACE2* mice without tamoxifen administration [UBC-*ACE2*, (n = 2)]; 2) infected UBC-*ACE2* mice treated with tamoxifen (UBC-*ACE2* ^Tx^, n = 8); 3) uninfected UBC-*ACE2* mice treated with tamoxifen as a histological control [UBC-*ACE2* ^Tx^ (Uninfected), n = 2], 4) infected Rosa-*ACE2* mice without tamoxifen administration [Rosa-*ACE2*, (n = 2)]; 5) infected Rosa-*ACE2* mice treated with tamoxifen (Rosa-*ACE2* ^Tx^, n = 9); 6) infected wild-type mice treated with tamoxifen (WT, n = 9); and 7) uninfected WT mice treated with tamoxifen as a histological control (n = 2—see Supplementary Table S1).

### SARS-CoV-2 Infection

**FIGURE 1:**
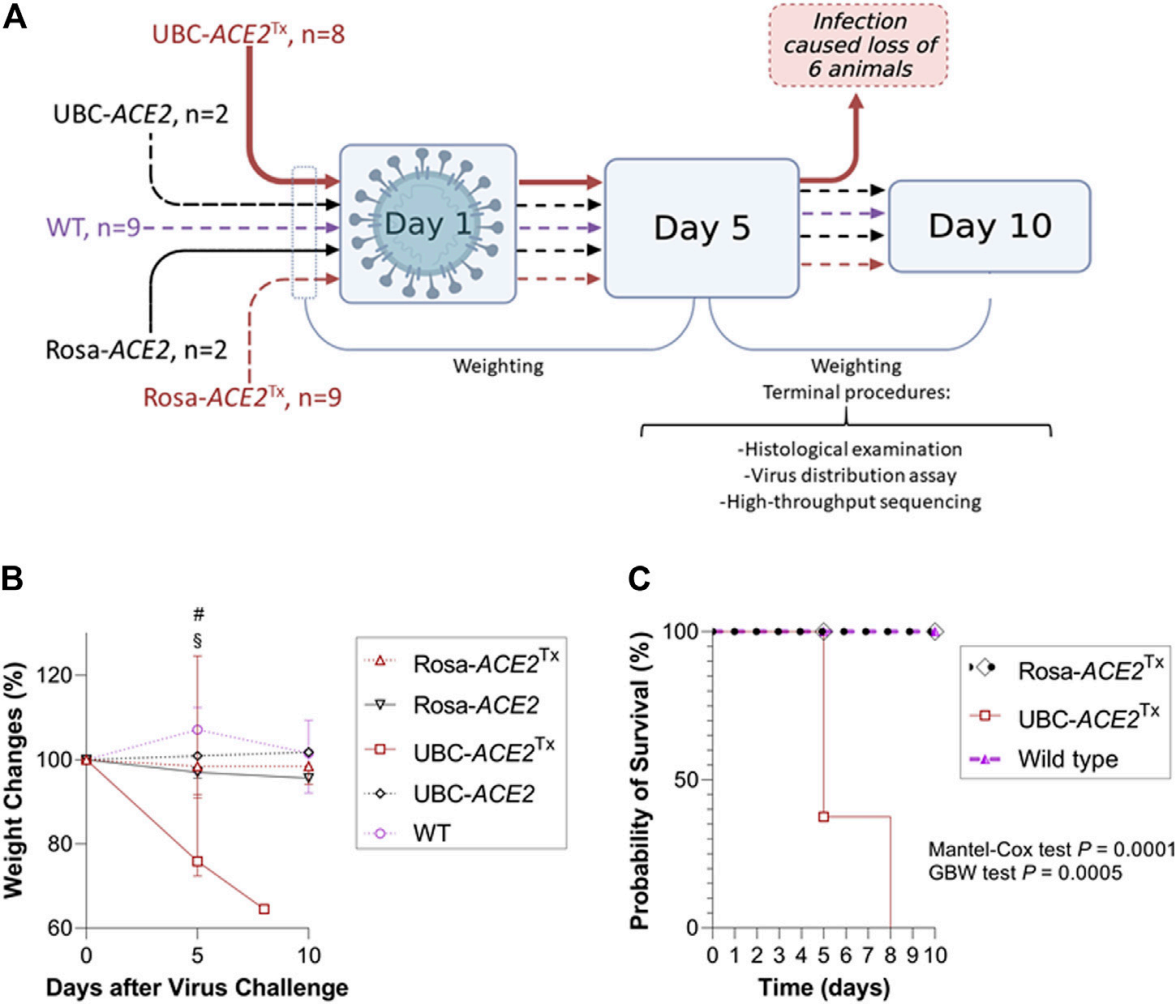
Evaluation of SARS-CoV-2 sensitivity in mice with Cre-dependent human (h)*ACE2* expression. **(A)** Graphical representation of the experimental design. All the mice in the UBC-*ACE2* ^Tx^ group died before the 10th day (Created with BioRender.com). **(B)** Dynamics of weight change after virus challenge; **(C)** Kaplan–Meier curve showing decreased survival in infected UBC-*ACE2* ^Tx^ mice.

All mice except 2 WT (Uninfected) and 2 UBC-*ACE2* ^Tx^ (Uninfected) mice (Figure 1A and Supplementary Table S1) were inoculated with SARS-CoV-2 (SARS-CoV-2/human/AUS/VIC01/2020) via the intranasal route at a dose of 4.5 lg FFU (focus-forming unit) as previously described (Agranovski et al., 2010). All experiments involving the use of live SARS-CoV-2 particles were carried out in a biosafety level-3 facility.

After viral inoculation, mice were observed daily. Weight was measured (NAVIGATOR™ NVT-2201, OHAUS, United States) before and after (5th and 10th days or just after death) inoculation. The 5th and 10th days were chosen as the endpoints for histological examination and postmortem studies of SARS-CoV-2 distribution and mutagenesis (Jiang et al., 2020) (Figure 1A).

### Evaluation of Virus Production

To determine viral biodistribution, the PCR-based determination of SARS-CoV-2 genome equivalent number was performed. Samples were diluted in Opti-MEM tissue culture medium (Gibco, United Kingdom) and homogenized with a TissueLyser LT tissue homogenizer (Qiagen, United Kingdom) according to the manufacturer’s instructions (the weight of tissue samples are presented in Supplementary Table S3). The samples were centrifuged for 5 min at 10,000 g, and the resulting supernatant was collected. The purification of total RNA from 100 μl samples was performed using a “RIBO-sorb” Extraction Kit (AmpliSens, Russia) according to the manufacturer’s recommendations. An internal control exogenous RNA (InterLabService, Russia) was added to each sample before purification to control the isolation, reverse transcription and PCR steps. Reverse transcription, followed by real-time PCR, was performed with a “Reverta-L” reagent kit (InterLabService, Russia) and Vector PCRrv-2019-nCoV-RG test system (SRC VB “Vector” Rospotrebnadzor, Russia) using the following primers: 5′-GTT​GCA​ACT​GAG​GGA​GCC​TTG-3' (forward), 5′-GAG​AAG​AGG​CTT​GAC​TGC​CG-3' (reverse) and 5′-FAM-TACACCAAAAGATCACATTGGCACCCG-BHQ1-3' (probe) according to the manufacturer’s recommendations. PCR was carried out using a RotorGene 6000 thermocycler (Qiagen, United States) with the program recommended in the PCR kit manufacturer’s manual. Sample Ct values were obtained in two fluorescence channels for viral cDNA and the internal control. The data obtained were analyzed using Rotor Gene 6000 Series Software 1.8.17.5. Ct values were recalculated into genome equivalents in samples based on the obtained standard dilution curve, with points from 10^−1^ to 10^−5^ of the initial viral stock used for infection. The standard curve had a high correlation coefficient (R^2^ > 0.99).

### Focus Formation Assay

To determine the concentration of live SARS-CoV-2 viral particles in the brain and lung tissues, FFU analysis of the Vero E6 cell line was performed (Supplementary Table S3). A cultured cell monolayer in 96-well plates was washed 2 times with tissue culture medium [DMEM with 300 μg/ml L-glutamine (BioloT, Russia), 100 U/ml penicillin G and 100 μg/ml streptomycin mix (Biolot, Russia), 10 μg/ml DEAE-dextran (Sigma, United States) and 5 μg/ml trypsin TPCK (Thermo FS, United States)]. In the wells of the 96-well plate, a 10-fold dilution series of the investigated viral suspension (tissue homogenate previously obtained with TissueLyser LT (Qiagen, United Kingdom)) was added. Then, 100 μl of each dilution of the virus was added to 4 wells. After that, 200 μl of cooled acetone (80%) was added for 15–20 min, then the acetone was removed and the plate was dried. Fifty microliters of a 1:1000 dilution of human monoclonal anti-SARS-CoV-2 antibody (COVID-19, 2019-nCoV) and nucleoprotein/NP monoclonal antibodies (FPZ0562, Fapon Biotech Inc., China) in PBS (Thermo FS, United States) was added to each well for 60 min. After that, the wells were washed two times and incubated with secondary rabbit antibodies against human IgG conjugated with HRP (AP309P, Sigma, United States) for 1 h. Signal detection was performed using an AEC substrate (Sigma, United States) according to the manufacturer’s protocol. The number of focus-forming units was counted in each well using an Olympus CK40 microscope (Olympus, United States). The virus titer was calculated as previously described (Sloutskin and Goldstein, 2014).

### Histological Analysis

For histological analysis, tissue samples were fixed in a 4% paraformaldehyde solution (Sigma, United States) for 48 h, followed by washing in water for 10 min. The samples were sequentially dehydrated in ethanol at increasing concentrations followed by processing with a xylol-paraffin mixture. Next, the samples were fixed in paraffin blocks using the Tissue-Tek VIP 6 AI Vacuum Infiltration Processor (Sakura Finetek, Japan) according to the manufacturer’s protocols. Paraffin sections 4–5 μm thick were prepared using a Microm HM 360 Automated Microtome (Marshall Scientific, United States). Sections were stained with hematoxylin and eosin using an automated Multiple Stainer Tissue-Tek Prisma tissue stainer (Sakura Finetek, Japan) according to the manufacturer’s protocol. Light-optical examination and microphotography were performed with an Imager Z1 microscope (Zeiss, Germany). An Olympus VS200 slide scanner was used to obtain overview images and ensure the same imaging parameters. Images were captured using a UPLXAPO 20×/0.80 lens (Olympus, United States).

### Immunohistochemical Analysis

For immunochemical analysis, slides of brain tissues were deparaffinized in xylene and rehydrated in a graded ethanol series. As an additional control, brain samples of SARS-CoV-2-inoculated hamsters were used. Antigen retrieval was conducted in a humidified chamber in a 37°C incubator using 0.05% trypsin (Sigma, United States) solution for 10 min. After cooling to room temperature, the slides were incubated overnight at 4°C with a nucleoprotein/NP monoclonal antibody (FPZ0562, Fapon Biotech Inc., China) diluted 1:500 in blocking buffer (1% BSA (BioloT, Russia) in TBS with Tween-20 (Abcam, United States)). After three washes in TBS (Abcam, United States), the slides were treated with a secondary Cy3-conjugated anti-human antibody (AB_2337718, Jackson ImmunoResearch, United States) diluted at 1:200 in blocking buffer. Additionally, slides were stained with DAPI and DABCO for nuclear visualization. Visualization was performed with an EVOS™ XL Core Imaging System (Thermo Fisher Scientific, United States).

### High-Throughput Sequencing and Data Analysis

To analyze virus nucleotide sequences, cDNA libraries were amplified from RNA samples using the ARTIС and SISPA protocols (Reyes and Kim, 1991; DNA Pipelines et al., 2020). Furthermore, the libraries were prepared with the NEBNext Ultra II DNA Library Prep Kit for Illumina (New England BioLabs, United States) and sequenced (2 × 300-bp cycles) using the Illumina MiSeq platform (Illumina, United States). Raw reads were first trimmed using cutadapt 3.4 (parameters: -error-rate 0.1 –times 10 –overlap 3 –minimum-length 20 –pair-filter = any) to remove adapter and primer sequences. fastp 0.20.0 (Chen et al., 2018) (parameters: -× -3-5 -r -l 20) was used to remove low-quality sequences and reads. The data were concatenated and *de novo* assembled with the coronaSPAdes module (Meleshko et al., 2021) from the SPAdes 3.15.2 assembler (Bankevich et al., 2012) using the default settings. Reads were mapped with BWA 0.7.17 (Li and Durbin, 2009) to the assembled sequence, and PCR duplicates were removed using Picard MarkDuplicates 2.25.4 (Toolkit, 2019) for SISPA-amplified libraries, followed by variant calling with iVar (Grubaugh et al., 2019). Variants were called at ≥50% in reads that were supported by a minimum of 10 reads and had a MAPQ ≥ 30. Only variants present in both ARTIC- and SISPA-amplified libraries were considered mutations.

### Statistical Analyses

Statistical analyses were performed in the R software environment v4.1.1. As many samples did not display a normal distribution (Shapiro–Wilk test and the Spiegelhalter test, “normtest” package) or variance equality (Levene’s test, “lawstat” package), nonparametric methods were used. For quantitative variables, the significance of the obtained results was determined using Kruskal–Wallis one-way analysis of variance and Dunn’s test of multiple comparisons using rank sums with the Benjamini–Hochberg procedure to decrease the false discovery rate (“dunn.test” package) as post-hoc analyses to identify significant differences in intergroup comparisons. GraphPad Prism 9.2.0 software was also used to create graphic material and for survival assessment via the Kaplan–Meier method together with log-rank (Mantel–Cox) and Gehan-Breslow-Wilcoxon (GBW) tests corrected by the Benjamini–Hochberg procedure. Each statistically processed sample contained at least two values. The significance threshold for all performed tests was set to *p* ≤ 0.05.

## Results

### Weight Loss After Infection

Among all SARS-CoV-2-infected groups, only UBC-*ACE2* ^Tx^ mice displayed a severe clinical picture of infection. For a more demonstrative representation of the relative weight changes shown in the plot, the following formula was used (Figure 1B and Supplementary Table S2):

$$Weight\;Change\;=\;\frac{100\;+\left(Weight\;on\;the\;Nth\;day\;-\;Weight\;on\;the\;0th\;day\right)}{Weight\;on\;the\;0th\;day}*100$$

A significant difference in mortality was observed between the UBC-*ACE2* ^Tx^ group and the other groups. In this group, we observed dramatic weight loss and rapid mortality. Weighing on the 5th day revealed an ∼24% weight decrease [*p* = 0.0021 and 0.0004, compared to the Rosa-*ACE2* ^Tx^ and WT groups, respectively (Figure 1B: # and §)] and an ∼35% decrease up to the moment the last animal died on the 8^th^ day.

Intergroup comparison of survival probability revealed that UBC-*ACE2* ^Tx^ animals had a significantly higher probability of death than mice from the Rosa-*ACE2* ^Tx^ and WT groups (in both comparisons *p* = 0.002 and 0.006, for the Mantel–Cox and GBW tests, respectively) (Figure 1C). Notably, although a portion of the animals were sacrificed and censored on the 5th day, based on the remaining animals, the Kaplan–Meier curve predicted 100% mortality by the 10th day. Neither the UBC-*ACE2* without tamoxifen nor the Rosa-*ACE2* ^Tx^ group showed any clinical sign of infection, mortality or statistically significant weight change, suggesting no dramatic infection process in these groups.

### SARS-CoV-2 Distribution in Organs

**FIGURE 2:**
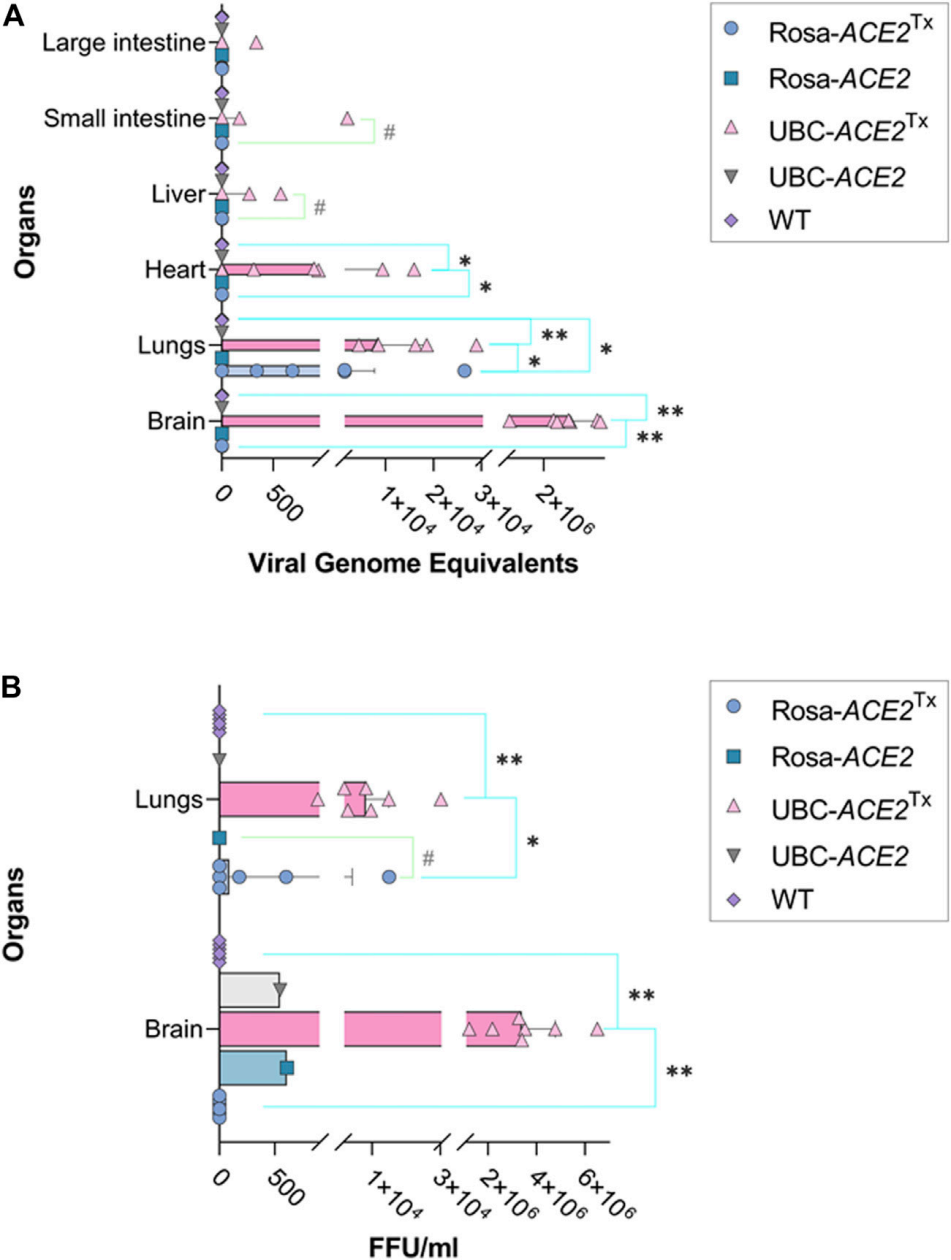
SARS-CoV-2 genome equivalent **(A)** and focus-forming unit **(B)** analyses in different samples of mouse organs on the 5th day of infection. **(A)** Significant differences between WT and UBC-ACE2^Tx^ and between Rosa-ACE2^Tx^ in heart and brain; significant differences between overall UBC-ACE2^Tx^, Rosa-ACE2^Tx^ and WT in lungs. **(B)** Significant differences between WT and UBC-ACE2Tx and between Rosa-ACE2^Tx^ in lungs and brain; *Note:* *p < 0.05, **p < 0.001, #*p* < 0.1.

Next, PCR and FFU analyses confirmed SARS-CoV-2 invasion and clarified its tissue distribution. On the 5th day, tissue samples were collected from all sacrificed and deceased animals. Intergroup comparisons of the obtained data were carried out only between the Rosa-*ACE2* ^Tx^, UBC-*ACE2* ^Tx^ and WT mice due to the small sample size in the remaining groups (Figure 2 and Supplementary Table S3).

Analysis of genome equivalents in lung samples revealed significant differences between all compared groups on the 5th day (Figure 2A). While the median level of viral genome equivalents in UBC-*ACE2* ^Tx^ mice was ∼8 times higher than that in Rosa-*ACE2* ^Tx^ mice (8465.05 vs. 1048.5, respectively, *p* = 0.0460), no virus was detected in the lungs of WT animals (*p* = 0.0004 and 0.0437 compared to UBC-*ACE2* ^Tx^ and Rosa-*ACE2* ^Tx^ mice, respectively). The same results were found during FFU analysis (Figure 2B): the UBC-*ACE2* ^Tx^ animals again had the highest amount of viable virus particles, which was ∼91 times more than the median value in the Rosa-*ACE2* ^Tx^ mice (8206.69 vs. 90.25, *p* = 0.0285). In contrast to the UBC-*ACE2* ^Tx^ and Rosa-*ACE2* ^Tx^ groups, there were no detected viable virus particles in lungs collected from WT animals. Similarly, the brain samples of UBC-*ACE2* ^Tx^ and Rosa-*ACE2* ^Tx^ animals were characterized by the presence of viral genome equivalents and viable virus particles. Only UBC-*ACE2* ^Tx^ mice showed the presence of viral genome equivalents in the heart, liver and intestine (Figure 2).

In the brain samples of #1 Rosa-*ACE2* and #2 UBC-*ACE2* mice, the presence of live viral particles was detected in the FFU assay, with values of 609.14 and 547.45 FFU normalized to the weight of the sample (Supplementary Table S3). During the PCR study of the genome equivalent, we did not detect the RNA of the virus in the tissue samples of these animals. This is because infected samples are inactivated at high temperature for PCR analysis, according to the standard protocol for working with pathogenic agents, which leads to the degradation of a part of the genomic RNA and to a decrease in the sensitivity of the method. Thus, the FFU assay is the more sensitive method of analysis in this case and allows the detection of even insignificant levels of infection in laboratory mice even in the absence of transgenesis, which was previously shown (Leist et al., 2020). However, in Rosa-*ACE2* and UBC-*ACE2* without tamoxifen administration, the FFU values were significantly lower than those in the experiment, and such an insignificant level of viral load was not pathogenic to laboratory mice.

The same PCR and FFU analyses were also performed on the 10th day (Supplementary Table S3). However, these data are not used for statistical estimation of virus distribution in organs due to the death of UBC-*ACE2* ^Tx^ mice and the absence of the viral genome in Rosa-*ACE2* ^Tx^ and WT samples.

### High-Throughput Sequencing Data Analysis

To analyze changes in the virus genome during mouse infection, we sequenced a virus from the original stock as well as postinfection virus samples from the brain and lung from #6 UBC-*ACE2* ^Tx^ as a representative of the UBC-*ACE2* ^Tx^ group. Here, using metatranscriptomic data, we obtained an almost complete genome (99% of reference genome SARS-CoV-2 coverage) with an average read depth of 277×. The sequencing reads were deposited in the NCBI SRA database under the accession number PRJNA780672. The assembled genomes were deposited in the GenBank database under accession number OL677207.1. None of the tissue-derived SARS-CoV-2 sequences contained any mutations relative to the original viral stock used for mouse infection.

### SARS-CoV-2-Induced Tissue Lesions

**FIGURE 3:**
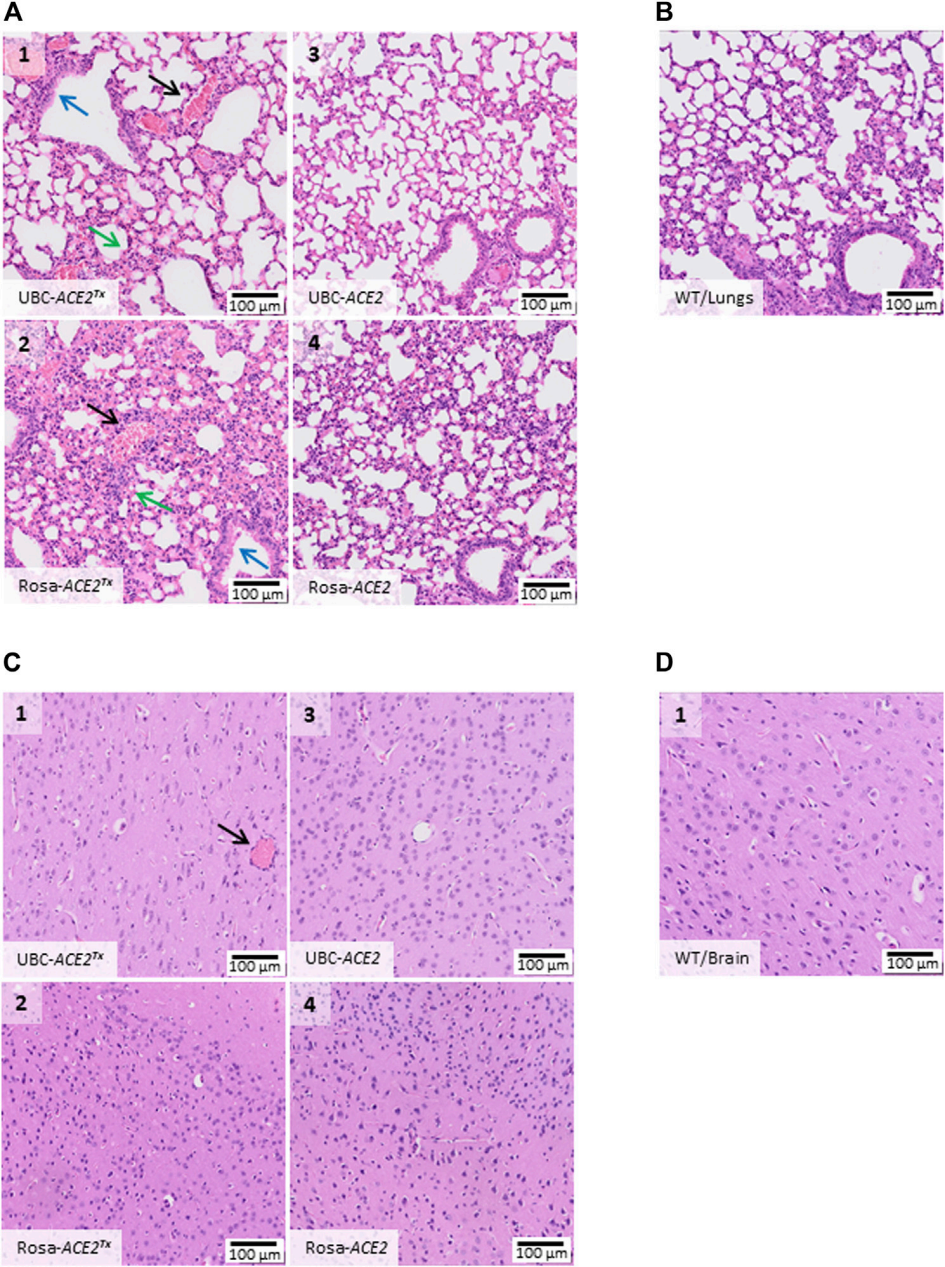
Histological alterations in organs on the 5th day after SARS-CoV-2 inoculation. **(A)** Hematoxylin/eosin histostained lung tissue samples from UBC-*ACE2* ^Tx^(A1), UBC-*ACE2* (A3), Rosa-*ACE2* ^Tx^ (A2) and Rosa-*ACE2* (A4) groups. **(C)** Hematoxylin/eosin histostained brain samples from the UBC-*ACE2* ^Tx^ (C1), UBC-*ACE2* (C3), Rosa-*ACE2* ^Tx^ (C2) and Rosa-*ACE2* (C4) groups. The pattern of pathological changes in UBC-*ACE2* ^Tx^(A1) and Rosa-*ACE2* ^Tx^ (A2) lung samples included the septal thickening of alveolar ducts and sacs caused by the diffuse hyperplasia of type II alveolar epithelial cells with mild lymphocytic infiltration and edema (green arrow), hyperplasia of the epithelium of bronchioles (blue arrow) and multiple sludges of erythrocytes in vessels (black arrow). Examination of brain samples revealed multiple sludges of erythrocytes (black arrow) only in brain vessels of UBC-*ACE2* ^Tx^ (C1) mice. In both the UBC-*ACE2* ^Tx−^ and Rosa-*ACE2* ^Tx-^infected groups, tissue lesions were detected only after tamoxifen administration. **(B)**, **(D)** WT mice displayed no damage in the studied organs.

Histological examination of animal tissue samples was conducted on the 5th day of infection (Figure 3). Lung samples from both UBC-*ACE2* and Rosa-*ACE2* mice without tamoxifen administration showed a normal tissue structure, as did the WT mouse group. Histological sections (Figures 3A3–4,B) show vessels, terminal respiratory bronchioles lined with ciliated columnar epithelium and alveolar ducts, sacs and alveoli lined with simple squamous epithelium that is interrupted with club cells on top of the septs. Unlike previous samples, UBC-*ACE2* ^Tx^ and Rosa-*ACE2* ^Tx^ mice showed pathological changes in lung tissues (Figures 3A1,2). These changes include the septal thickening of alveolar ducts and sacs caused by the diffuse hyperplasia of type II alveolar epithelial cells with mild lymphocytic infiltration and edema. In the bronchioles, the diffuse hyperplasia of the epithelium was observed. Moreover, lung tissue samples from UBC-*ACE2* ^Tx^ and Rosa-*ACE2* ^Tx^ mice were distinguished by multiple sludges of erythrocytes in vessels. This pattern of pathological changes corresponds to the proliferative phase of diffuse alveolar damage.

In contrast to lung samples, histological analysis of the brain samples did not reveal any significant changes among all groups (Figures 3C,D). Only multiple sludges of erythrocytes were observed in the vessels of UBC-*ACE2* ^Tx^ mice (Figure 3C1). UBC-*ACE2* ^Tx^ (Uninfected) and WT (Uninfected) mice treated with tamoxifen showed no pathological changes in tissue samples, indicating the absence of an effect of tamoxifen on the histological assay.

### Immunohistochemical Analysis

**FIGURE 4:**
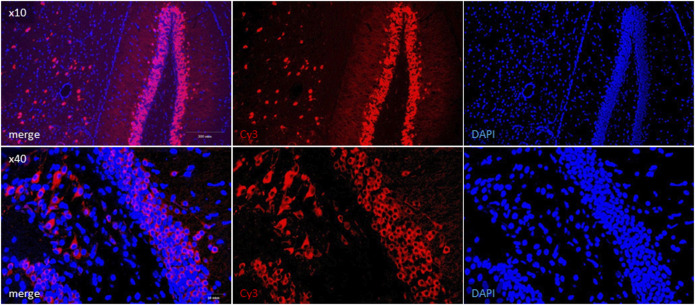
Immunohistochemical detection of SARS-CoV-2 N-protein in UBC-*ACE2* ^Tx^ brain samples. Viral N-protein was labeled with Cy3-conjugated secondary antibody (red field). Cell nuclei and DNA were stained with DAPI (blue field).

Since there is no consensus on SARS-CoV-2 neurotropism and neuroinvasion ability (Natoli et al., 2020; Pezzini and Padovani, 2020; Liu et al., 2021), we further visualized the presence of viral proteins in UBC-*ACE2* ^Tx^ brain samples. The most consideration was given to the dentate gyrus of the hippocampus due to its connection with the lateral entorhinal cortex that receives olfactory inputs via direct projections from the olfactory bulb (Woods et al., 2020). The virus has been shown to be located in neurons, which were distinguishable in slides as cells with soma and dendrites (Figure 4, Cy3). In mice without tamoxifen treatment (UBC-*ACE2*), infected WT mice and infected hamsters, specific signals indicating the presence of the SARS-CoV-2 N-protein were not observed (Supplementary Figures S1A–C).

## Discussion

In this article, we present the results of a study investigating the susceptibility of switch-on mice with inducible hACE2 expression to SARS-CoV-2 infection (Bruter et al., 2021). As the LoxP-STOP system provides temporal and spatial control of transgene expression, SARS-CoV sensitivity in ACE2-LoxP(STOP) mice might be precisely regulated by breeding with various Cre strains. Indeed, we show here that the UBC-*ACE2* and Rosa-*ACE2* strains are characterized by different severities of infection course. Although SARS-CoV-2 invasion was confirmed in both strains, only UBC-*ACE2* displayed acute weight loss and mortality. However, weight loss can be associated with either a viral infection or renin-angiotensin system since ACE2 needs to balance AngII levels. Its changes can lead to mouse feeding behavior alterations and consequently weight loss (Shorning et al., 2012). Unequal SARS-CoV-2 sensitivity might be related to different levels of transgene expression, consistent with previous data on UBC-driven and ROSA-26 locus-integrated promoter activity (Chen et al., 2011). Thus, two strains express the same Cre-ERT2 enzyme; however, the difference in its expression enables the resulting offspring to serve various research purposes.

When modeling a human disease using animal systems, we must estimate and consider the similarities and differences in the pathogenetic change patterns, for example, when developing antiviral therapy methods. To do this, we studied pathological changes in tissues during infection with the virus. Although SARS-CoV-2 causes severe pathology of the respiratory system, there is evidence that it affects the central nervous system in humans (Natoli et al., 2020; Pezzini and Padovani, 2020; Liu et al., 2021). For example, SARS-CoV-2 mRNA has been recovered from cerebrospinal fluid, suggesting that it can cross the blood–brain barrier (Moriguchi et al., 2020). Moreover, the virus has been found in brain tissues (Ren et al., 2021). It has also been shown that the penetration of the virus into the brain causes cerebral circulation (Helms et al., 2020). Some animal models do not show the presence of the virus in the brain. For example, the virus was not found in brain samples from the Syrian hamster. This has been described previously but was also confirmed in this study by immunohistochemistry. Our research showed that in the UBC-*ACE2* line, the virus penetrated directly into neurons in the gyrus dentate area. Considering that this part of the hippocampus receives olfactory inputs (Woods et al., 2020) and SARS-CoV-2 enters the central nervous system through the olfactory tract (Meinhardt et al., 2021), model mice can be used to study the interplay between COVID-19 and the nervous system. Although Cre-ERT2 is ubiquitously expressed, the analysis of SARS-CoV-2 distribution revealed the most significant viral load in brain and lung tissues. Apparently, these findings are related to the intranasal route of viral inoculation and may be similar to SARS-CoV-2 distribution in humans (Chen et al., 2021). We also provided evidence that mice without tamoxifen administration are resistant to SARS-CoV-2, suggesting that there is no undesired leakage in hACE2 expression; hence, the model is biosafe until an experiment is started.

It is also important to study whether the virus undergoes mutations during the mouse infection process. This information is necessary to understand in which studies these mice can be used, for example, antiviral drug research in which two factors are important: maintaining viral replication in mice and an adequate infection pattern. Both study types involve a search for differences in different viral strain activities, and thus, the use of certain cellular receptors is necessary for the correct modeling of the infection process. RNA-containing viruses are capable of rapid changes in their primary sequence, which is associated with their increased mutagenicity, leading to worldwide spread. For SARS-CoV-2, the use of a human ACE2 receptor can likely reduce its variability due to the lack of targeted selection for other types of this virus in other animal models. Indeed, no change in the viral sequence was detected during mouse infection.

## Data Availability

All data generated or analyzed during this study are included in this published article (and its supporting information files). The sequencing reads were deposited in the NCBI SRA database under the accession number PRJNA780672. The assembled genomes were deposited in the GenBank database under accession number OL677207.1.

## Supplementary Material

Supplementary Figure S1 Immunohistochemistry control. Immunohistochemical detection of SARS-CoV-2 particles in UBC-*ACE2* brain samples. The N-protein of SARS-CoV-2 (red field) was not observed in either WT mice **(A)** or UBC-*ACE2* without tamoxifen treatment **(B)**. Additionally, we did not find N-protein-positive signals in hamster brain samples, which is consistent with previous literature data related to SARS-CoV-2 infection patterns in these animals **(C)**. Cell nuclei were stained with DAPI (blue field).

Supplementary Table S1 The experimental design. In the table, the number of animals, their genotypes, experimental conditions and outcomes are summarized.

Supplementary Table S2 Changes in the weight of animals. The table discloses all primary and processed data concerning weight dynamics.

Supplementary Table S3 SARS-CoV-2 genome equivalent number and focus-forming unit analysis. Virus production is presented as genome equivalents measured for three groups of mice. Here, we describe additional data about virus detection in different organs (brain, lungs, heart, etc.). The results of FFU analysis are also shown.
