# Supplementary material for: The Tissue Distribution of SARS-CoV-2 in Transgenic Mice With Inducible Ubiquitous Expression of hACE2
Source: Front Mol Biosci. 2022 Jan 18;8:821506. doi: 10.3389/fmolb.2021.821506 (PMC8804232; doi:10.3389/fmolb.2021.821506)
Supplement: Supplementary file 3 [file Table1.DOCX]

***Supplementary table 1.*** *The experimental design.*

| **Genotype** | **Used label** | **Tamoxifen administration** | **SARS-CoV-2 infection** | **Sacrificed on the 5^th^ day (n)** | **Sacrificed on the 10^th^ day (n)** | **Deceased on the 5^th^ day (n)** | **Deceased on the 8^th^ day (n)** | **Overall** |
| --- | --- | --- | --- | --- | --- | --- | --- | --- |
| Rosa-*ACE2* | Rosa-*ACE2* | NO | Infection | 1 | 1 | 0 | 0 | 2 |
|  | Rosa-*ACE2*^Tx^ | Tx. administration |  | 6 | 3 | 0 | 0 | 9 |
| UBC-*ACE2* | UBC-*ACE2* | NO | Infection | 1 | 1 | 0 | 0 | 2 |
|  | UBC-*ACE2*^Tx^ | Tx. administration |  | 2 | 0 | 5 | 1 | 8 |
|  | UBC-*ACE2*^Tx^  (Uninfected) | Tx. administration | NO | 1 | 1 |  |  | 2 |
| Wild type | WT | Tx. administration | Infection | 6 | 3 | 0 | 0 | 9 |
|  | WT  (Uninfected) | Tx. administration | NO | 1 | 1 | 0 | 0 | 2 |
