# Supplementary figures and images for: The Tissue Distribution of SARS-CoV-2 in Transgenic Mice With Inducible Ubiquitous Expression of hACE2
Source: Front Mol Biosci. 2022 Jan 18;8:821506. doi: 10.3389/fmolb.2021.821506 (PMC8804232; doi:10.3389/fmolb.2021.821506)

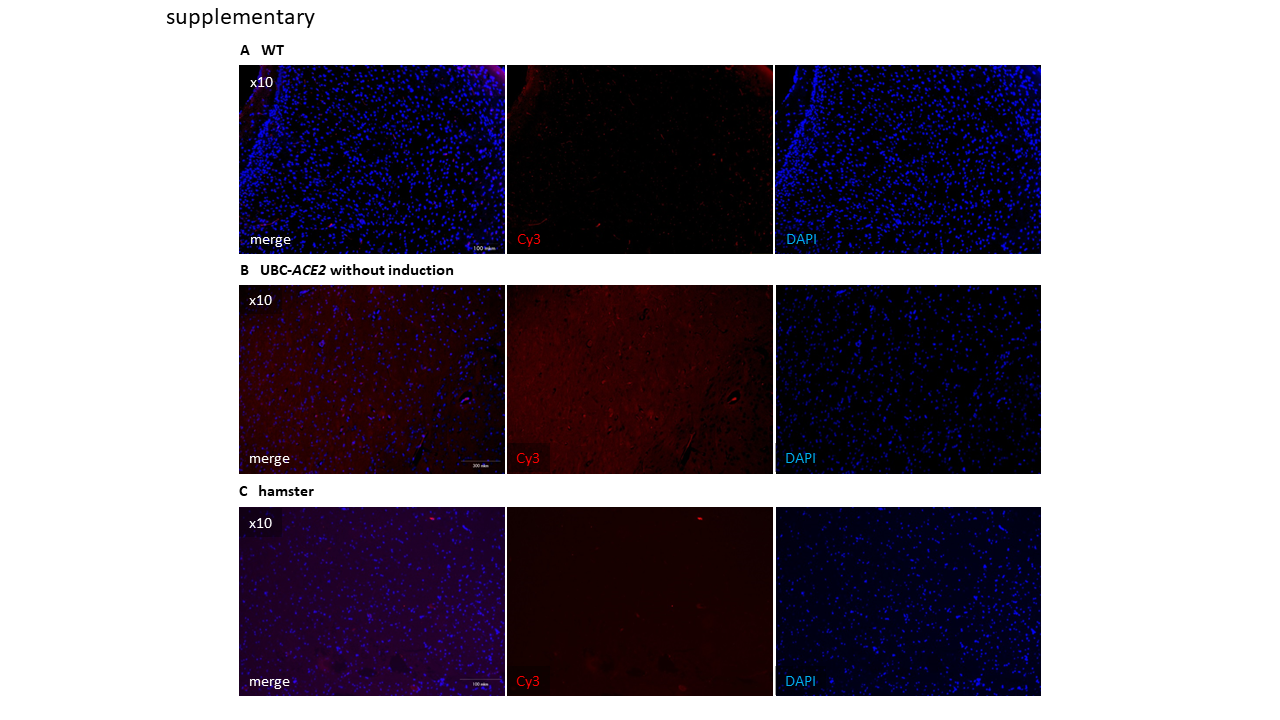

Supplement: Supplementary file 4 [file Image1.TIF]
